# Hierarchical Information Entropy System Model for TWfMS

**DOI:** 10.3390/e20100732

**Published:** 2018-09-24

**Authors:** Qiang Han, Deren Yang

**Affiliations:** 1 School of Computer Science and Engineering, North Minzu University, Yinchuan 750021, China; 2 Key Laboratory of Trustworthy Distributed Computing and Services (Ministry of Education), Beijing University of Posts and Telecommunications, Beijing 100871, China; 3 School of Science, Ningxia Medical University, Yinchuan 750021, China

**Keywords:** Trustworthy Workflow Management System, information entropy system model, fluctuation theorem, dissipative structure system, the second law of thermodynamics, system engineering, service computing, software engineering

## Abstract

Under the infrastructure of three gradually deepening layers consisting of System, Service and Software, the information entropy of the Trustworthy Workflow Management System (TWfMS) will evolve from being more precise to more undetermined, due to a series of exception event X occurring on certain components (ExCs), along with the life cycle of TWfMS, experienced in its phased original, as-is, to-be, and agile-consistent stages, and recover, more precisely again, by turning back to the original state from the agile-consistent stage, due to its self-autonomous improvement. With a special emphasis on the system layer, to assure the trustworthiness of WfMS, this paper firstly introduces the preliminary knowledge of the hierarchical information entropy model with correlation theories. After illustrating the fundamental principle, the transformation rule is deduced, step by step, followed by a case study, which is conducive to generating discussions and conclusions in the different research areas of TWfMS. Overall, in this paper, we argue that the trustworthiness maintenance of WfMS could be analyzed and computational, through the viewpoint that all the various states of TWfMS can be considered as the transformation between WfMS and its trustworthiness compensate components, whose information entropy fluctuate repeatedly and comply with the law of the dissipative structure system.

## 1. Introduction

**Figure 1:**
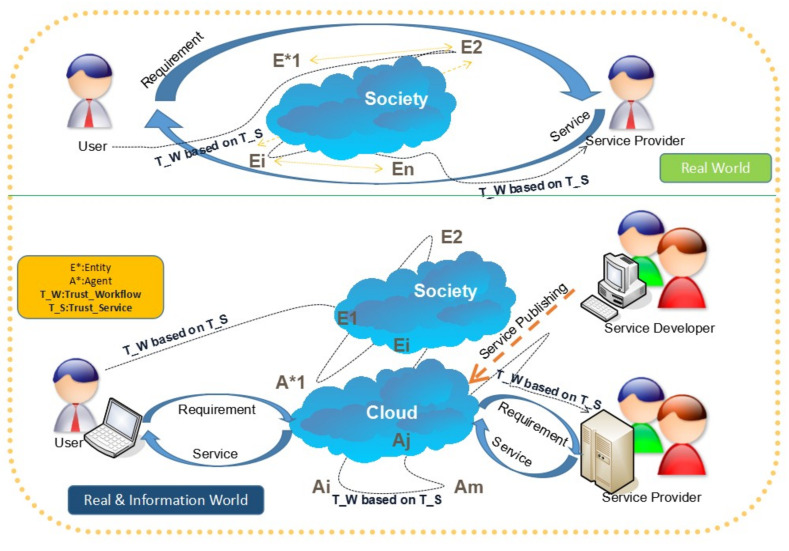
The trustworthy workflow of services in the Real and Information World.

As one of the chaotic effective metrics of the member systems that belongs to the workflow management system (WfMS), information entropy reflects the uncertainty of its trustworthiness, covered with gradually deepening layers from top to bottom, i.e., system engineering [1], service computing [2], and software engineering [3,4]. Under the iterative process, the entities in the real world and their relationships would evolve into more complex entities, and with the emergence of the Internet, their relations present new hybrid features that are mixed with the environment of the real world (origin) and the information world (migrated). When in doubt, in order to detail and summarize the complex evolution progress of WfMS, we must rely on a multidisciplinary view to carry out the relevant research works of WfMS, so that the trustworthy workflow of the service layer, which is between the system layer and software layer, can be verified and validated by the users in Figure 1. Just as ourselves feeling, the Entity (E*) and Agents (A*) are intertwined with each other, covering the real world and information world with the Trust_Workflow (T_W) based on the Trust_Service (T_S), into business processes among the users and service developers or providers. What needs to be particularly pointed out is that the Trust_Service (T_S) is encapsulated by the various instances of society and its digital form, i.e., cloud computing. Obviously, the Real and Information world in the lower part of Figure 1 have experienced a deep evolution far from the purely original Real World, without digitization present in the upper part of Figure 1, which consists of proxies with a single type, i.e., the Entity type. While the services were transferred from Service Providers to Users through Entities, a belief network was created that underlies the workflow consisting of the services. Therefore, the Trust_Workflow (T_W) was generated based on the Trust_Service (T_S). Progressively, with the digitalization evolution in the Real World, some entities undertake mechanized repetitive operations. These are selected out and changed into intelligent agents, which results in the emergence of the Real and Information World. As we know, compared with the original Real World, whatever the functions of the Real and Information World, the T_W in the Real and Information World should be maintained at the same level or at a higher level than the original Real World. Hence, we should conduct further research on the T_W from the top layer, i.e., system layer and its middle layer can focus on the service, and those on the bottom layer should focus on the software. As the early stage of this work, in this paper, we put the emphasis on the system layer in advance.

In order to reflect the complex evolutionary phenomenon mentioned above, this paper constructs an information entropy system model according to the dissipative structure relationship between states of WfMS via the interfaces connecting the components, which assures the trustworthiness of WfMS addressing special exception events on certain components of the WfMS, i.e., we proposed and argued the dissipative structure relationship on the quantities relationship of the information entropy of WfMS and those additional trustworthiness assure components regarding the same exception events, namely, that the former generates those events, and the latter eliminates them as thoroughly and fluently as possible. 

The remainder of this paper consists of five sections: Section 2 presents the motivation inspiring our research works; Section 3 discusses related research work on information entropy methods regarding WfMS; Section 4 illustrates the hierarchical information entropy system models for TWfMS on preliminary knowledge, fundamental principle, and transformation rules respectively; Section 5 gives the case study on the course timetabling problems from the case-based reasoning (CBR) system viewpoint; and Section 6 summarizes future research areas, with discussions and conclusions.

## 2. Motivation

**Figure 2:**
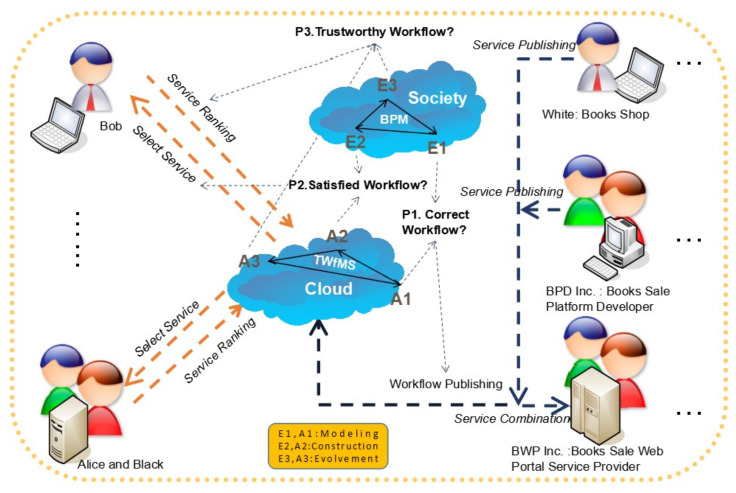
The application scenario of Trustworthy Workflow Management System (TWfMS) for ordering books.

Based on the trustworthy workflow of services in Figure 1, we can recognize that the ultimate trust information of the workflow will be a mix of the entities in the real world and the agents in the information world, which create a belief network between the users and the service developer, as well as the service providers. For example, while the stakeholders above engage with the belief network to tackle their business process management (BPM) in scenarios about book publishing, sales, and purchase activities, they should also resolve the following important problems illustrated in Figure 2.

In Figure 2, almost all the elements in Figure 1 are initialized in the application scenario for the order books workflow, through the automation of the business process. At first, when we temporarily ignore specific software development and service computing details in Figure 2, regardless of the existence of service computing tasks, a system viewpoint can assist us in realizing that all the initialized elements in Figure 2 already make up one digitized dissipative structure system, which continues to realize its negentropy process through the absorption and digestion of low-information-entropy, while, at the same time, it discharges high-information-entropy. In the other words, if we take “White: Books Shop” as an example, the low-information-entropy for White is the information on the electronic books or paper books from the authors by computer editors or printing houses. On the contrary, the high-information-entropy for White is the information of a books volume’s contents, which can be assembled by the “BWP Inc” on the “BPD Inc” platform. Furthermore, these Book Shops will compete with regard to their low-information-entropy, and cooperate with the Book Sale Platform Developers or Web Portal Service Providers, like “BPD Inc” or “BWP Inc” to make a contribution to their common high-information-entropy, i.e., the information of Workflow Publishing. Obviously, negentropy is realized through a similar phenomenon in which the entropy increase is suppressed on Entities in the society, as well as on Agents in Cloud, concurrently. Hence, users like Bob, Alice, and Black can tell that their requirements are always met through the TWfMS in Figure 2.

Additionally, with the emergence of software engineering, the information entropy of software behavior trustworthiness will ultimately determine whether the whole workflow system is trustworthy or not to users. This means system information entropy is determined by the software information entropy. Moreover, by addressing the flexible and scalable needs of traditional software engineering, users no longer require local ownership of the software. With the software service engineering emergence, service-oriented information entropy gradually inserts itself into the gap between the system and software behavior information entropy as a fundamental infrastructure and, finally, setting up the hierarchical information entropy model for TWfMS.

Therefore, when the workflow publishing service is assembled by BWP Inc. in Figure 2, users such as Bob, Alice, and Black should make decisions to select their satisfied workflow service (i.e., Problem 2, P2) through the service ranking given by other users’ evaluation on their feeling of the workflow trustworthiness (i.e., Problem 3, P3) based on the workflow correctness (i.e., Problem 1, P1) assisted by the workflow modelling function provided by E1 or A1 and the construction function provided by E3 or A3.

In general, all the behaviors of the system, service, and software of TWfMS in Figure 2 have evident characteristics that imply that the workflow information entropy is the negentropy of the business process dissipative structure system entropy, and that it transfers the trustworthiness of TWfMS to users. Hence, under such a scenario, we have sufficient motivation to research the hierarchical information system model for TWfMS to reveal its trustworthiness evolution law. As the first step in carrying out this research work, we plan to study system layer of the hierarchical information entropy model firstly in this paper.

## 3. Related Work

One of the most significant intelligence of BPM is the business process capability of any single enterprise in Figure 1 and Figure 2, that can be multiplied with the other enterprises’ services, so as to provide an amplified capability to its users, which means, in the view of its users, the capability of the single enterprise is not limited by its local resources, but depends on the computing performance of the whole WfMS. Obviously, the critical factor which influences the virtual capability of a single enterprise, based on the whole WfMS, is the fact that all of the enterprises have a unified trustworthiness framework which includes each other, otherwise, any single enterprise could not provide an amplified capability to its users. By addressing the problem of trust in each other in such a distributed computing environment, accompanied with the merging blockchain technology [5,6], its potential to support the Trust_Workflow (T_W) in Figure 2 can assist those enterprises in assembling their business process with an amplified capability by the delivery of a trust distributed storage certification. This is because its potential could drastically change the environment by challenging the joint design and a lack of mutual trust, which hampers the broader uptake of the interorganizational BPM.

With the trend of blockchain technology, sooner or later, researchers realized that the transference from an agent in the blockchain to another agent is information entropy, i.e., the producer agent supplies are high-entropy to the consumer agent, which are seen as low-entropy by the latter. In a similar dissipative business process, the producer agent also abstracts a certain volume of low-entropy for themselves, when compared to upper-level agents. Meanwhile, all of the agents’ structures in the whole WfMS are maintained in a stabile status with mutual trust. The phenomenon above is called the negentropy realizing process [7,8] of the dissipative structure system [9,10,11].

Inspired by the dissipative structure system theory and blockchain technology, we can consider the WfMS as a self-organizing dissipative structure system based on blockchain technology. Against this background, the difference between traditional dissipative structure system and WfMS is that the former is a type of dissipative structure system in which physicality is meaningful. On the contrary, the latter is a type of dissipative structure system in which information is meaningful. Therefore, to promote the degree of automation and controllability of the former, the latter is a mirror of the former. While the former consumes materials or energy to maintain their physical entropy structure in a loop, the latter consumes data to maintain their information entropy structure in a loop with as little material or energy as possible, in order to support the indispensable operations of the latter system, i.e., cloud computing center of IT enterprises. Hence, to a certain extent, the pressure to mirror sufficient data from the physical dissipative structure system into the information dissipative structure system, by using as little material or energy as possible, in which the materials consumed have been one the main motivations for keeping to Moore’s law [12], and the energy consumed is limited to Landauer’s principle [13,14].

Along with such a research view as above, we will overview the related works from the system, service, and software layers on the WfMS, respectively, through a continuous conversion chain from the physical entropy of the system to the information entropy of the system, service, and software, based on a trustworthy distributed database which was created by blockchain.

Firstly, on the system aspect, by introducing the applicability of choosing between models and designing hydrological networks for data collection, the authors of [15] illustrated the application of the principle of maximum entropy (POME). Just as what the authors of [16] present, Shannon presented the fundamental mathematical theory of communication to make a contribution to information theory, mainly for the technical problems, and addressed the semantic and effectiveness problems, such as the communication problem in hydrological networks [15]. Hence, due to the complex essential features in the system aspect, there are a series of hard problems for information science researchers [17,18]. While the software system can simulate, define, and control the business processes of the real world at a multilevel of granularity, the authors of [19] introduced a series of research works based on original and new research results on complex systems (CS) and fractional dynamics, as well as specific topics illustrating the broad impact of entropy and information theory-based techniques.

Secondly, on the service aspect, for optimizing the workflow, the authors of [20] presented an abstract model of visualization and inference processes, usually by making the transformation of the data spaces (referred to as alphabets) correspond to the reduction of maximal entropy. Addressing the selection of the best combination of clouds to meet the application requirements, the authors of [21] introduced an entropy-based method to quantify the most reliable workflow deployments and apply an extension of the Bell–LaPadula multilevel security model, to address the application security requirements, finally optimizing the deployment in terms of its entropy. With regards to the security of mobile cloud computing, the authors of [22] developed a Personalized Search scheme over encrypted data with efficient and secure Updates (PSU), which takes advantages of both the searchable symmetric encryption (SSE) and searchable public-key encryption (SPE) techniques.

As an entrance to user interface (UI) learning, the specification language is a critical element for the requirement of understanding the semantics of the workflow. The authors of [23,24] extended a specification language, SWSpec, with consideration to formal logic, so as to introduce some effective theorems in SWSpec, including a new compositional proof system and compliance checking technology.

Thirdly, on the software aspect, commonly, the propagation of software faults is caused by incorrect architecture designs in complex software, as a result of reflecting and deriving from the complex relationship in the real world. By modelling software fault propagation, the authors of [25] explored the interactions between fault propagation dynamics and software parameters. On the contrary, the authors of [26] presented a multilevel formation model for complex software systems, which can generate more realistic structural properties by organizing more realistic software networks. 

Corresponding to the references above, which mainly focus on the system and service model for the trustworthiness of WfMS, the authors of [27,28,29] made research works on the relative software model as an instantiation case of the system and service model, based on computer hardware. Especially on the software performance optimization model, the authors of [27] leveraged distribution entropy and its difference in an approximate Pareto front as a new objective space, in order to evaluate its individual environmental fitness, which is the metric used in updating the archive and selecting the global best solutions. On the software test optimization model, the authors of [28] demonstrated an approach to optimizing the software testing process by taking it as a Markov decision process based on the cross-entropy method; on the software resource optimization model, the authors of [29] proposed a resource evaluation model based on entropy optimization and dynamic weighting, which can filter the resources that satisfy user Quality of Service (QoS) and system maximization by a goal function and the constraints of maximum entropy and the entropy increase principle, so as to achieve optimal scheduling and satisfied user QoS.

Overall, the ultimate aim of almost all theories, regarding information systems, is to assist stakeholders in analysis, and design a perfect information system to meet the requirement of the users. To this end, the authors of [30] proposed the kernel concept family of the System Development Life Cycle (SDLC), and gave a completed approach to achieving the aim. Workflow management technology is a comprehensive advanced technology set to support the kernel concept of SDLC, and the authors of [31] described the ActionWorkflow^TM^ approach as a design methodology and associated computer software for the support of work in organizations.

In contrast to the related works summarized above, from the total goal to assure the trustworthiness of workflow, the proposed model in this paper separated the whole workflow system into two independent parts: that one is the original workflow components set, and another is the trustworthiness assurance components set. Due to the complex correlation of the two component sets, we abstract their correlation into the hierarchical information entropy system model by ignoring relatively minor influence parameters on the trustworthiness of workflow. Hence, the model proposed in this paper has relatively high robustness to adapt to the multivariable original, as-is, to-be, and agile-consistent stages of a workflow survival environment.

## 4. Information Entropy System Model for TWfMS

In line with the motivation and related works discussed above, as a special type of Quality Management System (QMS) [32] for the workflow, WfMSs experience multiple loops consisting of re-engineering and/or reorganizations [33] with the emergence of statistic mechanics [34] based on the behavior of business processes, and they converge into a certain type of information entropy. These processes of loops improve the WfMS into a high-level order, with a relatively low entropy of information systems, from a low-level order with a high entropy of information systems in the long term, along with consistently maintaining its trustworthiness, as defined by the stakeholders of WfMS [33], i.e., resulting in a dissipative structure system. This cycle, which covers the entire life of the WfMS, is also known as the resilience engineering (RE) [35] model, and it is based on our prior research [33]. We will put forward this work to illustrate its information entropy system model in this section for TWfMS. 

Based on the RE model for the WfMS [33], we illustrate our information entropy system model equations regarding TWfMS according to the standard WfMC reference model with the extension of methods and mechanisms, which is the fundamental goal of RE [33]. 

Considering that the implementation of RE for WfMS is imposed on the WfMS service components [36] in the middle stage, in this section, we firstly introduce the preliminary knowledge with the information entropy system model definition for the WfMS. Secondly, as the fundamental principle of WfMS RE, to assure its trustworthiness as perceived by users, we provide the fundamental quantity calculation principle, including relative constraints regarding to the information system entropy model definition of the WfMS. Thirdly, in order to implement TWfMS, the transformation rules based on the preliminary knowledge and fundamental principle are presented, as a guideline to develop TWfMS.

### 4.1. Preliminary Knowledge

**Figure 3:**
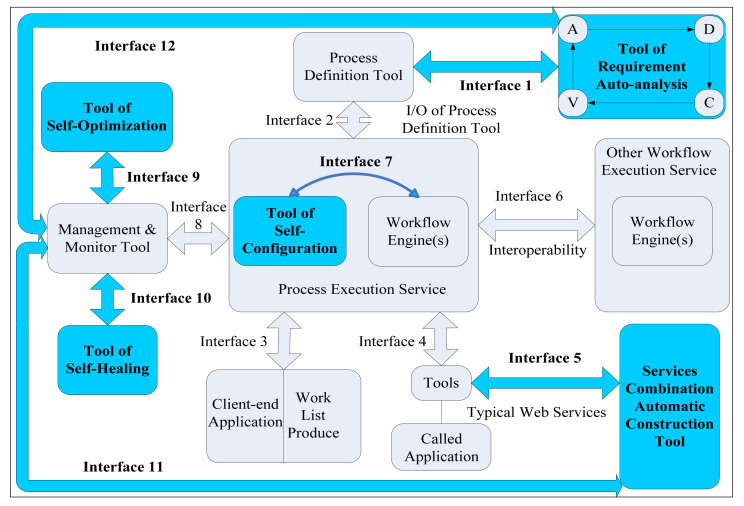
The reference model for TWfMS with RE.

Not just as System of Systems oriented (SoS-oriented), but also from the view of the dissipative structure system, the complex system software, a formal representation of the mechanisms and methods revealing the internal principles of WfMS trustworthiness, is imperative for the design, development, and maintenance tasks covering the entire WfMS life cycle. While it continues to consume high-entropy information from the supplier, it also produces low-entropy information. In our prior research, we presented a reference model for trustworthy WfMS (TWfMS) [33,36], as illustrated in Figure 3, inspired by the RE concept. As indicated in Figure 3, we expand the WfMC reference model from interfaces #0 and #6–#11. In the following paragraphs, we explain the information entropy model of system for TWfMS in terms of each of these interfaces.

A special entropy production on information system is derived from its role of non-equilibrium thermodynamics [37]. The “compensation” N in a certain cyclic process is defined by Equation (1)–(5), where T is the heat reservoir whose temperature is θ, dQr is the heat transferred between the systems, dQrT is the differential of a state function S (called the “entropy”), and dQ′ is called “uncompensated heat”, respectively, in the following conditions:

when the cyclic process is a reversible cycle, i.e., Equation (1):

(1) N≡−∫dQrT=0;

when the cyclic process is a reversible or irreversible cycle, i.e., Equation (2):

(2) N≡−∫dQT≥0;

when the cycle has a reversible path connecting two arbitrary equilibrium states 1 and 2, and an irreversible one connecting the same two states, i.e., Equation (3):

(3) N+∫(1)(2)dQT=S(2)−S(1);

when the cycle is seen as an infinitesimally arbitrary process, i.e., Equation (4):

(4) dS+dN=dQT;

when the cycle is faced by a dimensional requirement, i.e., Equation (5):

(5) dN=dQ′T.

Then, just as with the viewpoint of the authors of [37], what is the nature of N and how can we quantify it? The information entropy S is called the “negentropy” of the dQT [23] and, according to the statement, goes by the name of the second principle of thermodynamics [38] (Equation (6)):

(6) $$S\geq \frac{\Delta Q}{T}.$$

Additionally, in a statistically-based interpretation of thermodynamics, the directly linked entropy of the number of molecular configuration of W (the thermodynamic weight) of the system [39] is Equation (7):

(7) S=KlnW.

Then, after the first mention of “Negentropy” by Leo Szilard in 1929, Erwin Schrödinger applied “Negentropy” in biology, and made an argument that organisms rely on negative entropy to survive [40].

(8) $$\frac{dS}{dT}=\frac{d_{i}S}{dT}+\frac{d_{e}S}{dT},$$

where dS means the change of entropy of the whole system (e.g., in this paper, refer to the WfMS) and the dS is determined by the two elements, $d_{i}S$ and $d_{e}S$, in which $d_{i}S$ means the production of entropy from the inside of the whole system (e.g., in this paper refer to WfMS) and $d_{e}S$ means the exchange of entropy from the outside of the whole system, in other words, the environment (e.g., in this paper, refer to the implementation of the components which assure the trustworthiness of WfMS). This means that, at any time, because $d_{i}S\geq 0$, when $\left(d_{e}S\leq 0\right)\&\left(\left|d_{e}S\right|\geq d_{i}S\right)$, then dS≥0, (e.g., the increase of trustworthiness of the WfMS). Otherwise, dS<0 (e.g., the decrease of trustworthiness of the WfMS). Based on the theories above, we can give the following information entropy model of the system for TWfMS (Figure 3).

### 4.2. Fundamental Principle

To note the definition in the following hierarchical information entropy system model for TWfMS, and to be consistent with Equations (1)–(8), we declaim that $\left(\frac{R}{C}\int_{\left(WfMS-\#\mathrm{source}\right)}^{\left(WfMS-\#\mathrm{destination}\right)}E\left(c/o/h/n/r/\ldots /x/\ldots \right)Cs\right)$ as $d_{i}S$, $\left(\overset{WfMS-\#\mathrm{destination}}{\underset{WfMS-\#\mathrm{source}}{N}}(\#source)\right)$ as $d_{e}S$, and (S(WfMS−#destination)−S(WfMS−#source)) as dS, thus, if we assume all the change of entropy occurred in the same period of time dT, we can simplify Equation (8) as $dS=d_{i}S+d_{e}S$. Then, we have

(9) $$\begin{array}{l} dS=d_{i}S+d_{e}S=\left(\frac{R}{C}\int_{\left(WfMS-\#\mathrm{source}\right)}^{\left(WfMS-\#\mathrm{destination}\right)}E\left(c/o/h/n/r/\ldots /x/\ldots \right)Cs\right)+\left(\overset{WfMS-\#\mathrm{destination}}{\underset{WfMS-\#\mathrm{source}}{N}}(\#source)\right)\\ =\left(\sum_{i=1}^{ExCs}p\left(WfMS\_ExCs_{i}\right)\ln p\left(WfMS\_ExCs_{i}\right)\right)\\ -\left(\begin{array}{l} \sum_{i=1}^{ExCs}p\left(\overset{WfMS-\#destination}{N}{\left(\#\mathrm{source}\right)}_{i}\right)\ln p\left(\overset{WfMS-\#destination}{N}{\left(\#\mathrm{source}\right)}_{i}\right)\\ -\sum_{i=1}^{ExCs}p\left(\underset{WfMS-\#source}{N}{\left(\#\mathrm{source}\right)}_{i}\right)\ln p\left(\underset{WfMS-\#source}{N}{\left(\#\mathrm{source}\right)}_{i}\right) \end{array}\right)\\ =\left(S(WfMS-\#\mathrm{destination})-S(WfMS-\#\mathrm{source})\right) \end{array}$$

Furthermore, we declaim that the #destination/#source is a selection from the interface number in Figure 3, with the scope of 1–12; E(c/o/h/n/r/…/x/…)Cs counts on certain unemployed components by varying exception events in the runtime of WfMS, i.e., the WfMS experiences those exception events from the state represented by #source, and is promoted or recovered to the state represented by #destination through the implementation of the components, which assure the trustworthiness of WfMS according to special exception events on certain components, i.e., exception event X (ExCs). For example, parameter configuration (EcCs), performance optimization (EoCs), healing data (EhCs), requirement analysis (ErCs), and so on.

Moreover, in analogy to the Boltzmann constant [39], we argued that a similar constant $\frac{R}{C}$ in the hierarchical information entropy model has the means of the investment resource, including the man-month per standard component for a certain WfMS. Additionally, the information entropy of ExCs can be represented as $\left(\frac{R}{C}\int_{\left(WfMS-\#\mathrm{source}\right)}^{\left(WfMS-\#\mathrm{destination}\right)}E\left(c/o/h/n/r/\ldots /x/\ldots \right)Cs\right)\Rightarrow S=-\left(\sum_{i=1}^{ExCs}p\left(WfMS\_ExCs_{i}\right)\ln p\left(WfMS\_ExCs_{i}\right)\right)$, which means from the view of statistical mechanics [41], in all the exception events that occurred on WfMS from the state represented by #source, there is an ExCs amount of exception event X, and the probability of the *i-th* exception event X is $p\left(WfMS\_ExCs_{i}\right)$. Then, from the state of the #source to the state of the #destination, there is an $\left(\frac{R}{C}\int_{\left(WfMS-\#\mathrm{source}\right)}^{\left(WfMS-\#\mathrm{destination}\right)}E\left(c/o/h/n/r/\ldots /x/\ldots \right)Cs\right)$ amount of physical entropy occurring, for there is an ExCs amount of exception event X in the information entropy view. From the state of #source to the state of #destination, there is $S=-\left(\sum_{i=1}^{ExCs}p\left(WfMS\_ExCs_{i}\right)\ln p\left(WfMS\_ExCs_{i}\right)\right)$ amount of information entropy occurring for the same reason.

On the contrary, to realize a reversed cycle for WfMS or, in other words, to implement trustworthy WfMS (i.e., TWfMS), we should eliminate the information entropy of $S=-\left(\sum_{i=1}^{ExCs}p\left(WfMS\_ExCs_{i}\right)\ln p\left(WfMS\_ExCs_{i}\right)\right)$ as accurately as possible. Then, we attempt to promote or recovery the WfMS to their trustworthy state represented by #destination through the implementation of the components in Figure 3, which assure the trustworthiness of WfMS, i.e., $\left(\overset{WfMS-\#\mathrm{destination}}{\underset{WfMS-\#\mathrm{source}}{N}}(\#source)\right)$. Concerning the internal structure of the isolated component N for compensating the information entropy of $S=-\left(\sum_{i=1}^{ExCs}p\left(WfMS\_ExCs_{i}\right)\ln p\left(WfMS\_ExCs_{i}\right)\right)$, as implemented by system, service, or software method by a group of normal components, it also corresponds to unemployed components that emerge out to solve the i-th exception events X with a probability of $p\left(\underset{WfMS-\#source}{N}{\left(\#\mathrm{source}\right)}_{i}\right)$ before it starts to tackle those exception events X, and it also has corresponding components remaining unemployed and unused to solve those exception events X with a probability of $p\left(\overset{WfMS-\#destination}{N}{\left(\#\mathrm{source}\right)}_{i}\right)$, after it has tackled those exception events X. Hence, their difference $\left(\begin{array}{l} \sum_{i=1}^{ExCs}p\left(\overset{WfMS-\#destination}{N}{\left(\#\mathrm{source}\right)}_{i}\right)\ln p\left(\overset{WfMS-\#destination}{N}{\left(\#\mathrm{source}\right)}_{i}\right)\\ -\sum_{i=1}^{ExCs}p\left(\underset{WfMS-\#source}{N}{\left(\#\mathrm{source}\right)}_{i}\right)\ln p\left(\underset{WfMS-\#source}{N}{\left(\#\mathrm{source}\right)}_{i}\right) \end{array}\right)$ means the real work of N is to compensate the information entropy of $S=-\left(\sum_{i=1}^{ExCs}p\left(WfMS\_ExCs_{i}\right)\ln p\left(WfMS\_ExCs_{i}\right)\right)$, and their difference in Equation (9) means the change of entropy of WfMS, i.e., (S(WfMS−#destination)−S(WfMS−#source))≥0.

Therefore, we can make the assumption that if there are a certain amount of exception events X, the larger is (S(WfMS−#destination)−S(WfMS−#source)), and the more trustworthiness gained by us. Thus, some constraints on this fundamental principle can be made so that $(a):T_{stte}\left(WfMS\right)=T\left(WfMS-\#1\right)$ means the original version of WfMS-#1 owns the lowest trustworthiness (refer to trustworthiness on the software trusted threshold entropy [33]). In this paper, we prefer a trustworthiness on the system trusted threshold entropy of WfMS ($T_{stte}\left(WfMS\right)$), then, all the following versions of WfMS-#n (n > 1) should own a higher trustworthiness than WfMS-#1. $(b):T_{site}\left(WfMS\right)=T\left(WfMS-\#2\right)$ means the first improved version of WfMS-#2 owns the system trusted initialization trustworthiness entropy of WfMS ($T_{site}\left(WfMS\right)$). Then, normally, all the following versions of WfMS-#n (n > 2) should own a lower trustworthiness than WfMS-#2 for an unavoidable encounter with exception event X, resulting in the decrease of their trustworthiness.

$$\begin{array}{l} \left(c):\max\left(S(WfMS)\right)=S(WfMS-\#1\right)\\ \left(d):\min\left(S(WfMS)\right)=S(WfMS-\#2\right)\\ (e):T_{srte}\left(WfMS\right)\in \left[T\left(WfMS-\#2\right),T\left(WfMS-\#1\right)\right]\\ (f):T_{stte}\left(WfMS\right)\leq T_{srte}\left(WfMS\right)\leq T_{site}\left(WfMS\right) \end{array}$$

means that WfMS-#1 owns the maximum entropy in all the states of WfMS for the same reason as constraint (a) mentioned above, and that WfMS-#2 owns the minimum entropy in all the states of WfMS for the same reason as constraint (b) mentioned above. Hence, the system trusted runtime trustworthiness entropy of WfMS ($T_{srte}\left(WfMS\right)$) should be in the range of [T(WfMS−#2),T(WfMS−#1)].

On the other hand, generally speaking, there exists a certain correspondence between the behavior trustworthiness and system entropy of WfMS. That is, the larger the degree of confusion of the WfMS due to more exception events occurring, the lower the credibility of WfMS due to erroneous or misplace actions. However, concerning the complex structure of the WfMS in the system, service, and software layers, in some special conditions, the corresponding relationship is not necessarily established. Thus, we cannot give the calculation method of this corresponding relationship in this paper, something we plan to resolve in our future research.

$$(g):\sum_{i=1}^{ExCs}p\left(WfMS\_ExCs_{i}\right)=\sum_{i=1}^{ExCs}p\left(\overset{WfMS-\#destination}{N}{\left(\#\mathrm{source}\right)}_{i}\right)=\sum_{i=1}^{ExCs}p\left(\underset{WfMS-\#source}{N}{\left(\#\mathrm{source}\right)}_{i}\right)=1$$

means the sum the of probability of a type of exception event X occurring on a certain state where WfMS is 1, which is equal to the sum of the probability of the corresponding compensation actions reflected by the implementation of the components. This assures the trustworthiness of WfMS according to exception events X on certain components of WfMS. Either the sum of the probability of the corresponding compensation actions start to tackle the exception events X, or there are corresponding components that remain unemployed or unused to solve those exception events X.

### 4.3. Transformation Rule

Then, based on the definition discussed above, we illustrate the transformation rule of the hierarchical information entropy system model, step by step, on every interface, as follows:

(1) Interface #1 is linked to the requirement auto-analysis tool with the process definition tool, where the former consists of four components known as acquisition, decomposition, combination, and verification based on a Petri net (ADCV-PN); that is, we consider the ADCV-PN tools as an extension of and supplementary to the process definition tool [33]. As the supplementary components of WfMS and those dependent on TWfMS, we make the assumption that an error result probably exists in a certain entropy change, or lack of change, because of the exception event of the requirement analysis components (ErCs) through Interface #1.

(10) $$\begin{array}{l} \overset{WfMS-\#2}{\underset{WfMS-\#1}{N}}\left(\#1\right)+\frac{R}{C}\int_{\left(WfMS-\#1\right)}^{\left(WfMS-\#2\right)}ErCs\\ =-\left(\sum_{i=1}^{ErCs}p\left(\overset{WfMS-\#2}{N}{\left(\#1\right)}_{i}\right)\ln p\left(\overset{WfMS-\#2}{N}{\left(\#1\right)}_{i}\right)-\sum_{i=1}^{ErCs}p\left(\underset{WfMS-\#1}{N}{\left(\#1\right)}_{i}\right)\ln p\left(\underset{WfMS-\#1}{N}{\left(\#1\right)}_{i}\right)\right)\\ +\left(\sum_{i=1}^{ErCs}p\left(WfMS\_ErCs_{i}\right)\ln p\left(WfMS\_ErCs_{i}\right)\right)\\ =S(WfMS-\#2)-S(WfMS-\#1)\geq 0 \end{array}$$

(2) Interface #2 is linked to the process definition tool with the process execution service, where the former defines and validates the TWfMS generated by the tools of ADCV-PN; that is, we consider the process definition tool as a standard component of TWfMS with an absolute correctness. Indeed, the absolute correctness does not really exist, but, as the process definition tool is an original component of WfMS and an undependable component of TWfMS, we make the assumption that it is error-free. Then, based on that assumption, there would be no entropy change because of the exception event of the process definition components (EdCs) through Interface #2.

(11) $$\begin{array}{l} \overset{WfMS-\#3}{\underset{WfMS-\#2}{N}}\left(\#2\right)+\frac{R}{C}\int_{\left(WfMS-\#2\right)}^{\left(WfMS-\#3\right)}EdCs\\ =-\left(\sum_{i=1}^{EdCs}p\left(\overset{WfMS-\#3}{N}{\left(\#2\right)}_{i}\right)\ln p\left(\overset{WfMS-\#3}{N}{\left(\#2\right)}_{i}\right)-\sum_{i=1}^{EdCs}p\left(\underset{WfMS-\#2}{N}{\left(\#2\right)}_{i}\right)\ln p\left(\underset{WfMS-\#2}{N}{\left(\#2\right)}_{i}\right)\right)\\ +\left(\sum_{i=1}^{EdCs}p\left(WfMS\_EdCs_{i}\right)\ln p\left(WfMS\_EdCs_{i}\right)\right)\\ =S(WfMS-\#3)-S(WfMS-\#2)=0 \end{array}$$

(3) Interface #3 is linked to the process execution service with the “client-end application” and “work list produce”, where the latter assembles and calls for a remote client-end application and accepts batch tasks edited by the client-end user to produce a work list which aims to realize the reusability of some components in TWfMS−#1. Just like Interface #2, we make the assumption that it is error-free. Then, based on that assumption, there would be no entropy change because of the exception event of the “client-end application” and “work list produce” components (EeCs) through Interface #3.

(12) $$\begin{array}{l} \overset{WfMS-\#4}{\underset{WfMS-\#3}{N}}\left(\#3\right)+\frac{R}{C}\int_{\left(WfMS-\#3\right)}^{\left(WfMS-\#4\right)}EeCs\\ =-\left(\sum_{i=1}^{EeCs}p\left(\overset{WfMS-\#4}{N}{\left(\#3\right)}_{i}\right)\ln p\left(\overset{WfMS-\#4}{N}{\left(\#3\right)}_{i}\right)-\sum_{i=1}^{EeCs}p\left(\underset{WfMS-\#3}{N}{\left(\#3\right)}_{i}\right)\ln p\left(\underset{WfMS-\#3}{N}{\left(\#3\right)}_{i}\right)\right)\\ +\left(\sum_{i=1}^{EeCs}p\left(WfMS\_EeCs_{i}\right)\ln p\left(WfMS\_EeCs_{i}\right)\right)\\ =S(WfMS-\#4)-S(WfMS-\#3)=0 \end{array}$$

(4) Interface #4 is linked to the process execution service with the tools, where the latter assembles and calls for a remote application to search the typical web services. Just like with Interface 3#, we make the assumption that it is error-free. Then, based on that assumption, there would be no entropy change because of the exception event of the tools components (EtCs) through Interface #4.

(13) $$\begin{array}{l} \overset{WfMS-\#5}{\underset{WfMS-\#4}{N}}\left(\#4\right)+\frac{R}{C}\int_{\left(WfMS-\#4\right)}^{\left(WfMS-\#5\right)}EtCs\\ =-\left(\sum_{i=1}^{EtCs}p\left(\overset{WfMS-\#5}{N}{\left(\#4\right)}_{i}\right)\ln p\left(\overset{WfMS-\#5}{N}{\left(\#4\right)}_{i}\right)-\sum_{i=1}^{EtCs}p\left(\underset{WfMS-\#4}{N}{\left(\#4\right)}_{i}\right)\ln p\left(\underset{WfMS-\#4}{N}{\left(\#4\right)}_{i}\right)\right)\\ +\left(\sum_{i=1}^{EtCs}p\left(WfMS\_EtCs_{i}\right)\ln p\left(WfMS\_EtCs_{i}\right)\right)\\ =S(WfMS-\#5)-S(WfMS-\#4)=0 \end{array}$$

(5) Interface #5 is linked to the tools for communication on the called application of typical web services with an additional tool, the auto construction method for WfMS (ACM4WMS), based on services combination. That is, we consider the ACM4WMS tool an extension of, and supplementary to, the standard tools, linked to the process execution service module via Interface #4 [33]. As a supplementary component of WfMS and dependent on TWfMS, we make the assumption that an error probably exists as a result of a certain entropy change, or lack of change, because of the exception event of the service combination components (EsCs) through Interface #5.

(14) $$\begin{array}{l} \overset{WfMS-\#6}{\underset{WfMS-\#5}{N}}\left(\#5\right)+\frac{R}{C}\int_{\left(WfMS-\#5\right)}^{\left(WfMS-\#6\right)}EsCs\\ =-\left(\sum_{i=1}^{EsCs}p\left(\overset{WfMS-\#6}{N}{\left(\#5\right)}_{i}\right)\ln p\left(\overset{WfMS-\#6}{N}{\left(\#5\right)}_{i}\right)-\sum_{i=1}^{EsCs}p\left(\underset{WfMS-\#5}{N}{\left(\#5\right)}_{i}\right)\ln p\left(\underset{WfMS-\#5}{N}{\left(\#5\right)}_{i}\right)\right)\\ +\left(\sum_{i=1}^{EsCs}p\left(WfMS\_EsCs_{i}\right)\ln p\left(WfMS\_EsCs_{i}\right)\right)\\ =S(WfMS-\#6)-S(WfMS-\#5)\geq 0 \end{array}$$

(6) Interface #6 is linked to the process execution service with other workflow execution service, in order to improve the interoperability of WfMS assisted by outside workflow engine(s). Just like the similar reason above, we make the assumption that it is error-free. Then, based on that assumption, there would be no entropy change because of the exception event of other workflow execution service components (EwCs) through Interface #6.

(15) $$\begin{array}{l} \overset{WfMS-\#7}{\underset{WfMS-\#6}{N}}\left(\#6\right)+\frac{R}{C}\int_{\left(WfMS-\#6\right)}^{\left(WfMS-\#7\right)}EwCs\\ =-\left(\sum_{i=1}^{EwCs}p\left(\overset{WfMS-\#7}{N}{\left(\#6\right)}_{i}\right)\ln p\left(\overset{WfMS-\#7}{N}{\left(\#6\right)}_{i}\right)-\sum_{i=1}^{EwCs}p\left(\underset{WfMS-\#6}{N}{\left(\#6\right)}_{i}\right)\ln p\left(\underset{WfMS-\#6}{N}{\left(\#6\right)}_{i}\right)\right)\\ +\left(\sum_{i=1}^{EwCs}p\left(WfMS\_EwCs_{i}\right)\ln p\left(WfMS\_EwCs_{i}\right)\right)\\ =S(WfMS-\#7)-S(WfMS-\#6)=0 \end{array}$$

(7) Interface #7 is linked to the core work engine(s) component of the WfMS, with the additional self-configuration-parameter system RE tool for the WfMS (SCP4WMS) in the process execution service module. That is, we consider the SCP4WMS tools to be an extension of and supplementary to the process execution service module [33]. As a supplementary component of WfMS, and dependent on TWfMS, we make the assumption that an error probably exists as a result of a certain entropy change or lack of change, because of the exception event of the parameter configuration components (EcCs) through Interface #7.

(16) $$\begin{array}{l} \overset{WfMS-\#8}{\underset{WfMS-\#7}{N}}\left(\#7\right)+\frac{R}{C}\int_{\left(WfMS-\#7\right)}^{\left(WfMS-\#8\right)}EcCs\\ =-\left(\sum_{i=1}^{EcCs}p\left(\overset{WfMS-\#8}{N}{\left(\#7\right)}_{i}\right)\ln p\left(\overset{WfMS-\#8}{N}{\left(\#8\right)}_{i}\right)-\sum_{i=1}^{EcCs}p\left(\underset{WfMS-\#7}{N}{\left(\#7\right)}_{i}\right)\ln p\left(\underset{WfMS-\#7}{N}{\left(\#7\right)}_{i}\right)\right)\\ +\left(\sum_{i=1}^{EcCs}p\left(WfMS\_EcCs_{i}\right)\ln p\left(WfMS\_EcCs_{i}\right)\right)\\ =S(WfMS-\#8)-S(WfMS-\#7)\geq 0 \end{array}$$

(8) Interface #8 is the process execution service consisting of the core work engine(s) component of the WfMS with SCP4WMS via Interface #7, with the management and monitor tool. Just like the similar reason above, we make the assumption that the latter is error-free. Then, based on that assumption, there would be no entropy change because of the exception event of the management and monitor tool components (EmCs) through Interface #7.

(17) $$\begin{array}{l} \overset{WfMS-\#9}{\underset{WfMS-\#8}{N}}\left(\#8\right)+\frac{R}{C}\int_{\left(WfMS-\#8\right)}^{\left(WfMS-\#9\right)}EmCs\\ =-\left(\sum_{i=1}^{EmCs}p\left(\overset{WfMS-\#9}{N}{\left(\#8\right)}_{i}\right)\ln p\left(\overset{WfMS-\#9}{N}{\left(\#8\right)}_{i}\right)-\sum_{i=1}^{EmCs}p\left(\underset{WfMS-\#8}{N}{\left(\#8\right)}_{i}\right)\ln p\left(\underset{WfMS-\#8}{N}{\left(\#8\right)}_{i}\right)\right)\\ +\left(\sum_{i=1}^{EmCs}p\left(WfMS\_EmCs_{i}\right)\ln p\left(WfMS\_EmCs_{i}\right)\right)\\ =S(WfMS-\#9)-S(WfMS-\#8)=0 \end{array}$$

(9) Interface #9 is linked to the management and monitor tool with the self-optimization framework system for the WfMS (SOF4WMS) RE tools, which contributes to the TWfMS mechanism with the SCP4WMS tools. That is, we consider the SOF4WMS tool an extension of, and supplementary to, the original management and monitoring tool [33]. As a supplementary component of WfMS and dependent on TWfMS, we make the assumption that an error probably exists as a result of a certain entropy change, or lack of change, because of the exception event of the performance optimization components (EoCs) through Interface #9.

(18) $$\begin{array}{l} \overset{WfMS-\#10}{\underset{WfMS-\#9}{N}}\left(\#9\right)+\frac{R}{C}\int_{\left(WfMS-\#9\right)}^{\left(WfMS-\#10\right)}EoCs\\ =-\left(\sum_{i=1}^{EoCs}p\left(\overset{WfMS-\#10}{N}{\left(\#9\right)}_{i}\right)\ln p\left(\overset{WfMS-\#10}{N}{\left(\#9\right)}_{i}\right)-\sum_{i=1}^{EoCs}p\left(\underset{WfMS-\#9}{N}{\left(\#9\right)}_{i}\right)\ln p\left(\underset{WfMS-\#9}{N}{\left(\#9\right)}_{i}\right)\right)\\ +\left(\sum_{i=1}^{EoCs}p\left(WfMS\_EoCs_{i}\right)\ln p\left(WfMS\_EoCs_{i}\right)\right)\\ =S(WfMS-\#10)-S(WfMS-\#9)\geq 0 \end{array}$$

(10) Interface #10 is linked to the management and monitor tool with the self-healing model system for the WfMS (SHM4WMS) RE tools, which contributes to the TWfMS mechanism with the SCP4WMS and SOF4WMS tools. That is, we consider the SHM4WMS tool as an extension of, and supplementary to, the original management and monitoring tool [33]. As a supplementary component of WfMS and dependent on TWfMS, we make the assumption that an error probably exists as a result of a certain entropy change, or lack of change, because of the exception event of the healing data components (EhCs) through Interface #10.

(19) $$\begin{array}{l} \overset{WfMS-\#11}{\underset{WfMS-\#10}{N}}\left(\#10\right)+\frac{R}{C}\int_{\left(WfMS-\#10\right)}^{\left(WfMS-\#11\right)}EhCs\\ =-\left(\sum_{i=1}^{EhCs}p\left(\overset{WfMS-\#11}{N}{\left(\#10\right)}_{i}\right)\ln p\left(\overset{WfMS-\#11}{N}{\left(\#10\right)}_{i}\right)-\sum_{i=1}^{EhCs}p\left(\underset{WfMS-\#10}{N}{\left(\#10\right)}_{i}\right)\ln p\left(\underset{WfMS-\#10}{N}{\left(\#10\right)}_{i}\right)\right)\\ +\left(\sum_{i=1}^{EhCs}p\left(WfMS\_EhCs_{i}\right)\ln p\left(WfMS\_EhCs_{i}\right)\right)\\ =S(WfMS-\#11)-S(WfMS-\#10)\geq 0 \end{array}$$

(11) Interface #11 is connected to the management and monitoring tool with the ACM4WMS tool when the WfMS encounters local break points, whereby the WfMS trustworthiness can no longer be maintained by the management and monitoring tool, even with the assistance of the SCP4WMS, SOF4WMS, and SHM4WMS toolsets. In the context of the scenario described above, via Interface #11, the management and monitoring tool transfers the unsolved exceptional event by the SCP4WMS, SOF4WMS, and SHM4WMS toolsets, sequentially, to the ACM4WMS tool, in order to reconstruct the WfMS by searching for resources in the cloud. At such a time, we consider the WfMS as the beginning of local resilience engineering [33]. As a supplementary component of WfMS and dependent on TWfMS, we make the assumption that an error probably exists as a result of a certain entropy change, or lack of change, because of the exception event of the local resilience engineering components (ElCs) through Interface #11.

(20) $$\begin{array}{l} \overset{WfMS-\#5}{\underset{WfMS-\#11}{N}}\left(\#11\right)+\frac{R}{C}\int_{\left(WfMS-\#11\right)}^{\left(WfMS-\#5\right)}ElCs\\ =-\left(\sum_{i=1}^{ElCs}p\left(\overset{WfMS-\#5}{N}{\left(\#11\right)}_{i}\right)\ln p\left(\overset{WfMS-\#5}{N}{\left(\#11\right)}_{i}\right)-\sum_{i=1}^{ElCs}p\left(\underset{WfMS-\#11}{N}{\left(\#11\right)}_{i}\right)\ln p\left(\underset{WfMS-\#11}{N}{\left(\#11\right)}_{i}\right)\right)\\ +\left(\sum_{i=1}^{ElCs}p\left(WfMS\_ElCs_{i}\right)\ln p\left(WfMS\_ElCs_{i}\right)\right)\\ =S(WfMS-\#5)-S(WfMS-\#11)\geq 0 \end{array}$$

(12) Interface #12 is connected to the management and monitoring tool with the ADCV-PN tools when the WfMS encounters global break points, whereby the WfMS trustworthiness can no longer be sufficiently accurate by means of the ACM4WMS tool, even if all the resources in the cloud are traversed by means of the ACM4WMS tool. In the context of the scenario described above, via Interface #12, the management and monitoring tool finally transfers the exceptional event unsolved by the ACM4WMS tool to the ADCV-PN tools, in order to remodel the WfMS under user validation. At such a time, we consider the WfMS as the beginning of global resilience engineering [33]. As a supplementary component of WfMS and dependable on TWfMS, we make the assumption that an error probably exists as a result of a certain entropy change, or lack of change, because of the exception event of the global resilience engineering components (EgCs) through Interface #12.

(21) $$\begin{array}{l} \overset{WfMS-\#1}{\underset{WfMS-\#12}{N}}\left(\#12\right)+\frac{R}{C}\int_{\left(WfMS-\#12\right)}^{\left(WfMS-\#1\right)}EgCs\\ =-\left(\sum_{i=1}^{EgCs}p\left(\overset{WfMS-\#1}{N}{\left(\#12\right)}_{i}\right)\ln p\left(\overset{WfMS-\#1}{N}{\left(\#12\right)}_{i}\right)-\sum_{i=1}^{EgCs}p\left(\underset{WfMS-\#12}{N}{\left(\#12\right)}_{i}\right)\ln p\left(\underset{WfMS-\#12}{N}{\left(\#12\right)}_{i}\right)\right)\\ +\left(\sum_{i=1}^{EgCs}p\left(WfMS\_EgCs_{i}\right)\ln p\left(WfMS\_EgCs_{i}\right)\right)\\ =S(WfMS-\#1)-S(WfMS-\#12)\geq 0 \end{array}$$

## 5. Case Study

The author of [33] has introduced the application of WfMS addressing the course timetabling problems [42] from the case-based reasoning (CBR) system viewpoint. In this section, we will discuss a case study by using the CBR system for a university in China. Firstly, all the course timetabling business processes are managed by stakeholders consisting of students, teachers, the undergraduate teaching secretary, the director of the office of teaching, the dean of departments and schools, and the administrative staff of the department of education of the university. Therefore, just as we found, the original WfMS of CBR system existed in the business processes tackled and managed by those stakeholders with almost purely traditional personal and physical resources. Then, we can gain the original version of WfMS-#1 by acquisition. The decomposition based on a Petri net (AD-PN) toolsets indicates the lowest trustworthiness of WfMS with the highest information entropy, followed by the generation of the WfMS-#2 version by the combination and verification based on Petri net (CV-PN) tools, to indicate the highest trustworthiness of WfMS with the lowest information entropy. The related activities implemented from WfMS-#1 to WfMS#2 correspond to Equation (10) in Section 4. 

Secondly, through the process definition tool, assisted by the specification language with semantic formalization [18,19] on the CBR business, WfMS-#2 can be transformed into WfMS-#3 smoothly, without additional entropy needing to be eliminated. The related activities implemented from WfMS-#2 to WfMS-#3 correspond to Equation (11), with the condition of S(WfMS−#3)−S(WfMS−#2)=0 in Section 4. 

Next, when defined, the workflow instructions written by the specification language enter the control of the process execution service. This is read by the workflow engine(s) of the CBR system, the process execution service would call for the client-end application, and the users may deliver a work list by assembling the workflow instructions in a reuse pattern, e.g., in a university. This step means the CBR system running in the information center of the university would call for the data generated from the application of the department implemented by the software developed by the department or manually completed by the undergraduate teaching secretary. At the same time, the undergraduate teaching secretary can also deliver a work list by reusing the workflow instructions, for example, he/she may deliver a request about an undergraduate graduation qualification examination by reusing the instructions on the compulsory course score inquiry. Then, the process execution service can start the related activities implemented from WfMS-#3 to WfMS-#4, which correspond to Equation (12), with the condition of S(WfMS−#4)−S(WfMS−#3)=0 in Section 4.

If there are services which are not supplied by the university itself, but are necessary to complete the workflow, then, the CBR system would call for an application from outside of the university, e.g., searching the IEEE/ACM 2013 computing curriculum by a web crawler service. With such a background, the process execution service can start the related activities implemented from WfMS-#4 to WfMS-#5, which correspond to Equation (13) with the condition of S(WfMS−#5)−S(WfMS−#4)=0 in Section 4.

Furthermore, addressing the curriculum credit replacement for the exchange of undergraduate students studying abroad, the dean of the department would let the CBR system call for the service combination through the automatic construction tool by consulting with the corresponding dean of department or colleagues of the university abroad, which is normally completed by a traditionally personal method using a physical resource, via a mobile telephone or email, which has had rather low effectiveness in the last decade. Now, this is tackled by intelligent agents based on data mining on the big data of the text resources of the mass curriculum training program. With such a background, the additional tool will link to the ACM4WMS as a supplementary tool to start the related activities implemented from WfMS-#5 to WfMS-#6, which correspond to Equation (14) in Section 4.

As a mature cross-organization, WfMS, in this case, would encounter the collaborative work requirement by its peer WfMS, i.e., the CBR system would call for the undergraduate archives WfMS of the smart campus management information system in the same university, or the foreign students’ archive information from an overseas university. With such a background, the process execution service can start related activities implemented from WfMS-#6 to WfMS-#7, which correspond to Equation (15) with the condition of S(WfMS−#7)−S(WfMS−#6)=0 in Section 4.

Until this ordinal stage, the TWfMS has been created from WfMS-#1 to WfMS-#7 through 6 interfaces. If the CBR system can work smoothly by eliminating the exception events of the requirement analysis and service combination (ErCs, EsCs), then nothing needs to be done, and the trustworthiness of WfMS complies with the constraint condition (e) in Section 4. However, the unavoidable exception events of parameter configuration, performance optimization, and healing data (EcCs, EoCs, and EhCs) will result in the decrease of the WfMS trustworthiness. Then, the core workflow engine(s) can start the related activities implemented from WfMS-#7 to WfMS-#8, which correspond to Equation (16) in Section 4, to solve the problem of the EcCs. This is followed by the management and monitor tool, which starts the related activities implemented from WfMS-#8 to WfMS-#9, which correspond to Equation (17) with the condition of S(WfMS−#9)−S(WfMS−#8)=0 in Section 4. 

Moreover, the management and monitor tool also starts the related activities implemented from WfMS-#9 to WfMS-#10, and from WfMS-#10 to WfMS-11#, to solve the problem of the EoCs and EhCs, which correspond to Equations (18) and (19) in Section 4. Obviously, the EcCs stands for the functionalities of WfMS, e.g., to adapt to the limited classroom capacity with an ever-increasing number of students; the EoCs stands for the non-functionalities of WfMS, e.g., to adjust the venue to a new classroom that better suits the preference of the teacher and students; and the EhCs stands for the business transactions consistency of WfMS, e.g., to adapt to achieve more consistent teaching transaction log data with a uniform state.

Then, until this advanced self-autonomous stage, the TWfMS has been created from WfMS-#1 to WfMS-#11 through 10 interfaces. If the CBR system can work smoothly by eliminating the exception events of ErCs, EsCs, EcCs, EoCs, and EhCs, then nothing needs to be done, and the trustworthiness of WfMS always complies with the constraint condition (e) in Section 4, until condition (e) is violated by a serious exception event of EsCs, EcCs, EoCs, and EhCs (ErCs were not included as they have been eliminated via interface #1). However, these unavoidable exception events of service combination, parameter configuration, performance optimization, and healing data (EsCs, EcCs, EoCs, and EhCs) will result in the decrease of the trustworthiness of WfMS, then, the management and monitor tool will start the related activities implemented from WfMS-#11 back to WfMS-#5, which correspond to Equation (20) in Section 4, in order to solve the problem of local resilience engineering (LRE). Otherwise, if the LRE routine fails, the management and monitor tool will start the related activities implemented from WfMS-#12 back to WfMS-#1, which correspond to Equation (21) in Section 4, in order to solve the problem of global resilience engineering (GRE) in Section 4.

Hence, after the ordinal stage from WfMS-#1 to WfMS-#7, and the advance self-autonomous stage from WfMS-#7 to WfMS-#11, TWfMS maintains its trustworthiness by LRE or GRE, connecting the methods and mechanisms in its life cycle. This means that the CBR system can always assure its trustworthiness when it encounters ElCs or EgCs exception events, e.g., the revision or creation of new versions of undergraduate curriculum programs.

## 6. Discussions and Conclusions

### 6.1. Discussions

Through the case study in Section 5, we find that as indicated in the Equations (10–21), the Business Process Management System (BPMS) will evolve into a Workflow Management System (WfMS) by Computer Aided Software Engineering (CASE) experimental and development environment, via semi automation pattern mixed with manual intervention and, furthermore, the WfMS will ultimately evolve into Trustworthy WfMS (TWfMS), because of its continuously disappearing high-entropy information and gaining of low-entropy through the work of “compensation” N, which refers to the $d_{e}S$, i.e., the exchange of entropy from the outside of the whole system, in other words, the environment (e.g., in the Section 5, all of the exception events handled in the system consist of the emergency management works of the stakeholders, which implement the automated or artificial components, which assure the trustworthiness of WfMS with the computational expressions as:

$$(\overset{WfMS-\#\mathrm{destination}}{\underset{WfMS-\#\mathrm{source}}{N}}(\#source))=-\left(\begin{array}{l} \sum_{i=1}^{ExCs}p\left(\overset{WfMS-\#destination}{N}{\left(\#\mathrm{source}\right)}_{i}\right)\ln p\left(\overset{WfMS-\#destination}{N}{\left(\#\mathrm{source}\right)}_{i}\right)\\ -\sum_{i=1}^{ExCs}p\left(\underset{WfMS-\#source}{N}{\left(\#\mathrm{source}\right)}_{i}\right)\ln p\left(\underset{WfMS-\#source}{N}{\left(\#\mathrm{source}\right)}_{i}\right) \end{array}\right),$$

in Section 4.1).

**Figure 4:**
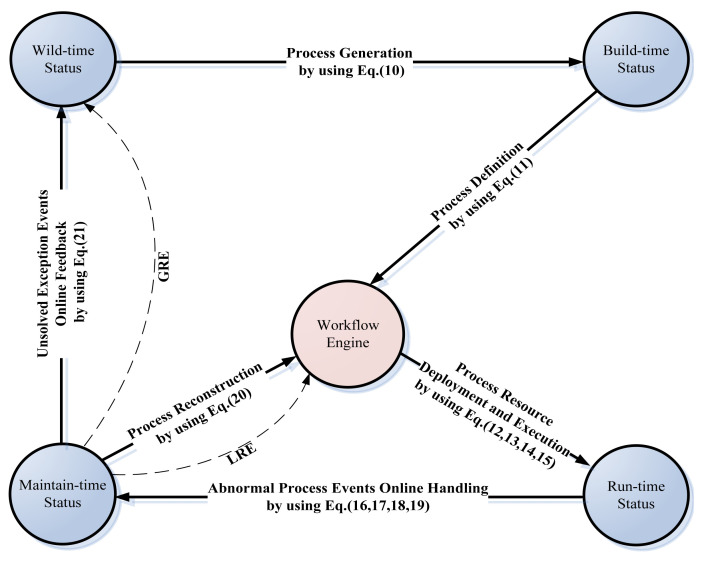
TWfMS state transition diagram.

In addition, while the TWfMS experiences continuous transformation via Equations (10–21), its status will also experience iterative Wild-time [43], Build-time, Run-time, and Maintain-time simultaneously, which is corresponding to the original, “as-is”, “to-be”, and “agile-consistent” stages in its life cycle, respectively, partially through the local resilience engineering (LRE) or the global resilience engineering (GRE) road map, as in Figure 4.

Thus, comparing with state-of-the-art achievement relative to BPMS or WfMS, the advantage of our proposals in this paper is that we divide and rule the principle for the transformation between WfMS and its trustworthiness compensate components, according to the second law of thermodynamics, because of the fact the information is negentropy of unemployment physics behavior.

Obviously, there is still a long way to go and hard work to be completed, in order to implement TWfMS using the proposals in this paper. As far as we know, addressing the gradually deepening self-adaptive and self-autonomous requirement to maintain the trustworthiness of WfMS, hyperparameters, machine learning, and search techniques [44], will play an important role in such a scenario described in Section 2. However, just as with the position the paper critically evaluated, genetic programming and hyper-heuristics should be embedded into the framework of large scale software development [45], e.g., TWfMS. Hence, due to there is no free lunch theorems for optimization [46], not only do we need traditional knowledge discovery in a database (KDD) process for extracting useful knowledge from Big Data [47], but we should also investigate the key technologies with interoperable interface specification standardization for developing TWfMSs.

### 6.2. Conclusions

Just like the author of [48] said many times, the “silver bullet” in software engineering does not exist. This means it is not realistic to strive to improve software development efficiency just by incremental Man-Months, for its inherent characteristics, i.e., that software is a mirror of the real world [49] with nonlinear complexity which is not yet fully controlled and understood by humans, although it is an artificial product. Therefore, plenty of classical artificial products and complex giant systems, with more complexity than the real world, have emerged recently, due to the linear, nonlinear, and even higher order complexity that has been woven into the life cycle of software by its artificial development processes, e.g., discovery process of critical nodes in road networks [50] with user-influenced behavior [51]. Thus, assuring the trustworthiness [52,53] of WfMS with the three characteristics of system, service, and software, is a problem that needs a solution urgently.

In order to address the measurement problem of WfMS trustworthiness, looking back at our prior research work, the authors of [54] proposed a software behavior trustworthiness measurement method as guidance for the following measurement algorithm [55]. Then, after a service-oriented trustworthiness measurement approach of components was presented for Business Process Re-engineering Application Server (BPRAS) [36], a relatively complete framework [56] of TWfMS has been described and integrated, with the more perfect software trustworthiness definitions [57] which were initially given by the authors of [54]. Furthermore, considering entropy as the main thread running through WfMS’s physics and informatics characteristics [33], from the view of a dissipative structure system, this paper proposed a hierarchical information entropy system model for TWfMS. This means that the deterministic entropy of the WfMS is always in a non-equilibrium state, even the state of being far from equilibrium [58,59,60,61], and that the aim of the users is used to maintain it in such a stable non-equilibrium state with current requirements. Alternatively, it encounters a new stable non-equilibrium state from the previous non-equilibrium state, due to the unavoidable continuous requirement changes [62,63,64].

In future research works, we plan to study the other two characteristics of service computing, the software engineering of TWfMS, and a novel algorithm for TWfMS trustworthiness based on the TWfMS framework mechanisms in an uncertain environment with incomplete software behavior test cases through the maximum-entropy model and its related applications [65]. We hope that the future research work can create a human-level control bridge connecting the hierarchical information entropy model of system, service, and software as a whole, using machine learning and artificial intelligence methods [66,67], so as to reveal essential features of the entropy of TWfMS, from physics to information sciences [68,69].

In summary, there is no denying the fact that as long as there are human activities, Business Process Management (BPM) will always exist, supported by BPM System (BPMS), and, underlying the progress of automation and informatization on BPMS, WfMS will always exist, whether we study it from the viewpoint of physical informatics or not, and with the convenience of service computing, the ultimately realized TWfMS functions by computer software and hardware will be continuously brought to both companies and home users in the foreseeable future.
